# Elevation of Hemoglobin A1c Increases the Atherosclerotic Plaque Vulnerability and the Visit-to-Visit Variability of Lipid Profiles in Patients Who Underwent Elective Percutaneous Coronary Intervention

**DOI:** 10.3389/fcvm.2022.803036

**Published:** 2022-02-03

**Authors:** Duanbin Li, Ya Li, Cao Wang, Hangpan Jiang, Liding Zhao, Xulin Hong, Maoning Lin, Yi Luan, Xiaohua Shen, Zhaoyang Chen, Wenbin Zhang

**Affiliations:** ^1^Department of Cardiology, Sir Run Run Shaw Hospital, College of Medicine, Zhejiang University, Hangzhou, China; ^2^Key Laboratory of Cardiovascular Intervention and Regenerative Medicine of Zhejiang Province, Hangzhou, China; ^3^Department of Cardiology, Haiyan People's Hospital, Jiaxing, China; ^4^Department of Cardiology, The Fourth Affiliated Hospital, College of Medicine, Zhejiang University, Yiwu, China; ^5^Department of Cardiology, The First Affiliated Hospital, College of Medicine, Zhejiang University, Hangzhou, China; ^6^Department of Cardiology, Union Hospital, Fujian Medical University, Fuzhou, China

**Keywords:** hemoglobin A1c, optical coherence tomography, lipid variability, plaque vulnerability, percutaneous coronary intervention

## Abstract

**Background:**

Increased plaque vulnerability and higher lipid variability are causes of adverse cardiovascular events. Despite a close association between glucose and lipid metabolisms, the influence of elevated glycated hemoglobin A1c (HbA1c) on plaque vulnerability and lipid variability remains unclear.

**Methods:**

Among subjects undergoing percutaneous coronary intervention (PCI) from 2009 through 2019, 366 patients received intravascular optical coherence tomography (OCT) assessment and 4,445 patients underwent the scheduled follow-ups within 1 year after PCI. Vulnerability features of culprit vessels were analyzed by OCT examination, including the assessment of lipid, macrophage, calcium, and minimal fibrous cap thickness (FCT). Visit-to-visit lipid variability was determined by different definitions including standard deviation (SD), coefficient of variation (CV), and variability independent of the mean (VIM). Multivariable linear regression analysis was used to verify the influence of HbA1c on plaque vulnerability features and lipid variability. Exploratory analyses were also performed in non-diabetic patients.

**Results:**

Among enrolled subjects, the pre-procedure HbA1c was 5.90 ± 1.31%, and the average follow-up HbA1c was 5.98 ± 1.16%. By OCT assessment, multivariable linear regression analyses demonstrated that patients with elevated HbA1c had a thinner minimal FCT (β = −6.985, *P* = 0.048), greater lipid index (LI) (β = 226.299, *P* = 0.005), and higher macrophage index (β = 54.526, *P* = 0.045). Even in non-diabetic patients, elevated HbA1c also linearly decreased minimal FCT (β = −14.011, *P* = 0.036), increased LI (β = 290.048, *P* = 0.041) and macrophage index (β = 120.029, *P* = 0.048). Subsequently, scheduled follow-ups were performed during 1-year following PCI. Multivariable linear regression analyses proved that elevated average follow-up HbA1c levels increased the VIM of lipid profiles, including low-density lipoprotein cholesterol (β = 2.594, *P* < 0.001), high-density lipoprotein cholesterol (β = 0.461, *P* = 0.044), non-high-density lipoprotein cholesterol (β = 1.473, *P* < 0.001), total cholesterol (β = 0.947, *P* < 0.001), and triglyceride (β = 4.217, *P* < 0.001). The result was consistent in non-diabetic patients and was verified when SD and CV were used to estimate variability.

**Conclusion:**

In patients undergoing elective PCI, elevated HbA1c increases the atherosclerotic plaque vulnerability and the visit-to-visit variability of lipid profiles, which is consistent in non-diabetic patients.

## Background

Coronary artery disease (CAD) has contributed to the cardiovascular disease being the leading cause of death around the world (1). Meanwhile, as a traditional risk factor, diabetes mellitus (DM) doubles or even triples the incidence of CAD (2).

DM is a disease of abnormal metabolism and characterized by chronic hyperglycemia. Of all the glycemia indicators, glycated hemoglobin A1c (HbA1c) has been a well-established one for the assessment of long-term glycemic levels (3). The American Diabetes Association (ADA) and the World Health Organization (WHO) recommend HbA1c ≥6.5% and 5.7–6.4% as the diagnostic cut-off points for diabetes and pre-diabetes, respectively (4). In diabetic patients, elevated HbA1c level has been confirmed to increase the risk of cardiac death, cardiovascular diseases, and strokes (5). Even in non-diabetic CAD patients, elevated HbA1c levels were also related to a raised risk of long-term mortality and myocardial infarction (MI) (6, 7). However, the mechanism by which HbA1c levels affect the prognosis of CAD patients remains unclear.

The increased plaque vulnerability is associated with dyslipidemia and has been identified as the leading cause of adverse cardiovascular events (8). Due to the high resolution (10–15 μm), optical coherence tomography (OCT) allows the quantitative assessment of vulnerability features in the coronary artery (9, 10). Thinner fibrous cap thickness (FCT), larger lipid cores, and more macrophage infiltration all indicate a greater vulnerability feature and an underlying poor prognosis (11). The visit-to-visit lipid variability has been verified as another strong predictor of adverse cardiovascular events for CAD patients (12, 13). Even in the general population, elevated lipid variability has also been shown to raise the incidence of all-cause death, myocardial infarction (MI), and strokes (14). The variability of several lipids has been identified to be associated with adverse cardiovascular events, including low-density lipoprotein cholesterol (LDL-C), high-density lipoprotein cholesterol (HDL-C), non-high-density lipoprotein cholesterol (non-HDL), total cholesterol (TC), and triglyceride (TG) (15–18). Some genetic-level evidence has indicated that glucose dysregulation may be associated with increased lipid variability (19, 20).

Due to the strong association between glucose and lipid metabolisms, elevated HbA1c may contribute to adverse cardiovascular events by increasing plaque vulnerability and the visit-to-visit lipid variability. However, these potential relationships remain unclear. Therefore, we conducted the current study to explore the influence of HbA1c on plaque vulnerability features and lipid variability.

## Methods

### Study Subjects

**Figure 1:**
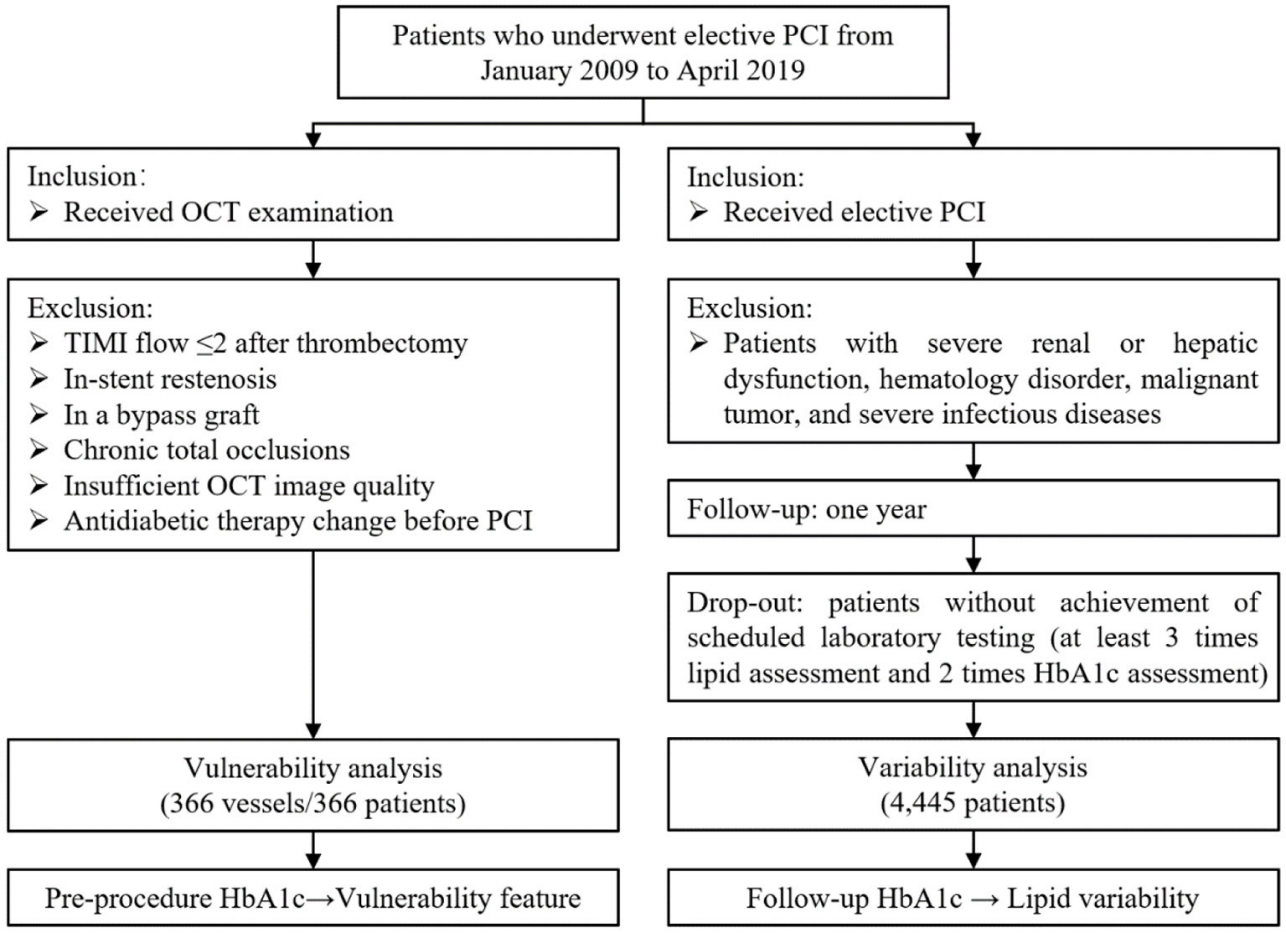
Flow chart of the current study.

In this retrospective cross-sectional study, patients who underwent elective PCI were eligible for screening from January 2009 through April 2019. The flow chart of the current study is shown in Figure 1. Patients were included if they received elective PCI and/or OCT examination. In the vulnerability feature analysis, subjects were excluded according to (i) culprit vessels with Thrombolysis in Myocardial Infarction (TIMI) flow ≤ 2; (ii) in-stent restenosis; (iii) bypass graft vessel; (iv) chronic total occlusion vessel; (v) insufficient OCT image quality; and (vi) antidiabetic therapy change before PCI. If the patient had multiple vessels examined by OCT, only the culprit vessel will be included. Besides, in lipid variability analyses, subjects were excluded if patients had severe renal or hepatic dysfunction, hematology disorder, malignant tumor, and severe infectious diseases. Patients who did not achieve scheduled laboratory tests (at least 3 times lipid assessments and 2 times HbA1c assessments during 1-year follow-up) were considered dropping out. Eventually, a total of 366 culprit vessels from 366 independent subjects were enrolled in the vulnerability feature analyses, and 4,445 patients were enrolled in the visit-to-visit lipid variability analyses. The ethical approval was obtained from the Medical Ethical Review Committee of Sir Run Run Shaw Hospital (NO. 20201217-36).

### The Assessment of HbA1c Levels

Pre-procedure and follow-up HbA1c levels were measured and recorded for analysis. According to the criteria of ADA and WHO, subjects were divided into 3 groups based on HbA1c levels of normal status (Tertile1: HbA1c <5.7%), pre-diabetes status (Tertile2: HbA1c 5.7-6.4%), and diabetes status (Tertile3: HbA1c ≥6.5%) (4). Pre-procedure HbA1c levels were used for vulnerability analysis, and average follow-up HbA1c levels were used for lipid variability analysis. In this study, all patients were recommended the regular HbA1c testing at 3rd, 6th, 9th, and 12th months after the PCI procedure. Enrolled patients should receive at least 2 times HbA1c assessments (interval at least 3 months) during 1-year follow-ups.

### The Acquisition of OCT Images

OCT examinations were conducted after intracoronary use of approximately 150 μg nitroglycerin. The current study employed the frequency domain OCT (C7-XR system, Saint Jude Medical, Westford, MA, USA). By using the non-occlusive flushing technique, the OCT imaging catheter was automatically pulled back and scanned for the morphology of culprit vessels at a speed of 20 mm/s. The obtained image was stored digitally. Off-line OCT image analysis was performed using proprietary OCT Review Software.

### The Vulnerability Assessment by OCT Examination

**Figure 2:**
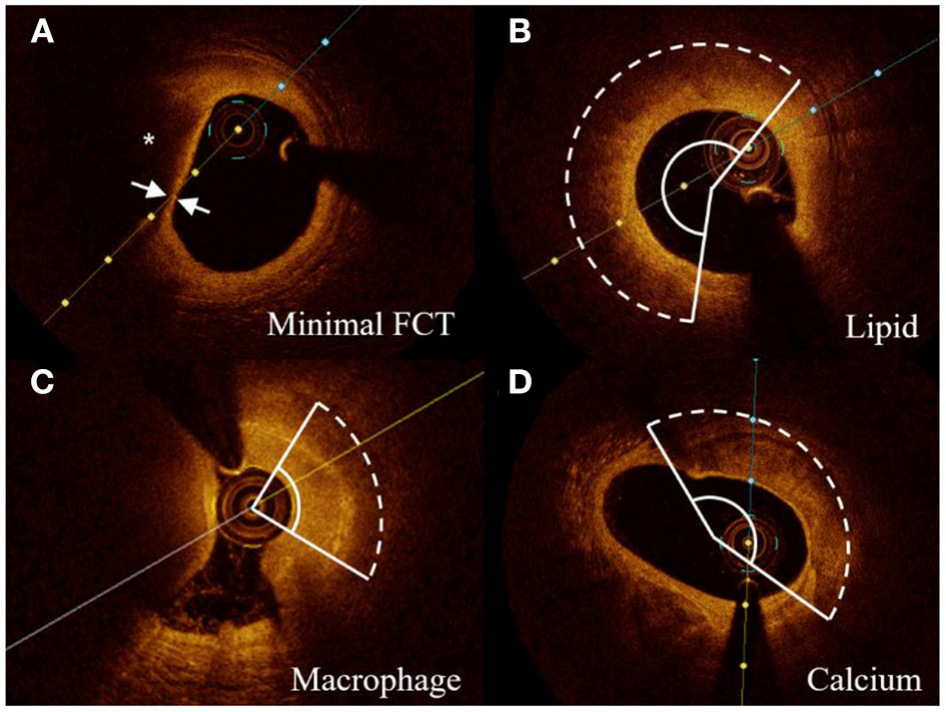
Representative cross-sectional OCT images. **(A)** Lipid core (*) and minimal FCT were detected. **(B–D)** The arcs of lipid accumulation, macrophage infiltration, and calcium deposition were measured in representative cross-sectional OCT images, respectively. FCT indicates fibrous cap thickness.

Vulnerable morphology features of culprit vessels were defined using previously established criteria (11). Representative OCT images of plaque features were shown in Figure 2. The minimal FCT was measured for three times, and a mean value was recorded. The lipid accumulation was a strong signal attenuation region with a signal-rich fibrous cap overlying. The macrophage infiltration was 1 or more signal-rich regions with sharp trailing attenuation that changed frame-by-frame. The calcium depositions were characterized by having poor signals and sharp borders. The angles of lipid accumulation, macrophage infiltration, and calcium deposition were analyzed every 1 mm with the mass center of lumens being angle points, and mean angles were calculated. The length of lipid accumulation, macrophage infiltration, and calcium deposition was computed as the total frame number of each finding multiplied by the distance between adjacent frames. To integrate the angle and length of vulnerability features, a volume index was calculated by mean angle × total length.

The vulnerability feature of culprit vessels was reviewed by 2 experienced interventional cardiologists who were blinded to the angiography and clinical presentation. Repeated measurements of 30 random OCT pullbacks were performed to determine intra-observer reproducibility of the same reader after 4 weeks following the initial measurement. An analysis of the consistency (inter- and intra-observer) was estimated by the intra-class correlation coefficient (ICC). A good reproducibility of inter- and intra-observer was verified on lipid arcs (ICC = 0.902, 0.917) and macrophage arcs (ICC = 0.875, 0.903), calcium arcs (ICC = 0.892, 0.912), and minimal FCT (ICC = 0.914, 0.927).

### The Assessment of Visit-to-Visit Lipid Variability

All subjects were recommended the lipid assessment scheduled at 1st, 3rd, 6th, 9th, and 12th months after the PCI procedure. Enrolled patients should receive at least 3 times lipid assessments during follow-ups. The blood sample was collected after fasting for more than 8 h. For a more comprehensive assessment of lipid variability, three different indices were employed: (i) the standard deviation (SD); (ii) the coefficient of variation (CV, calculated as SD/mean ×100%); (iii) variability independent of the mean (VIM, calculated as SD/mean^α^ ×100%) with α being regression coefficient based on the natural logarithm of SD and the natural logarithm of the mean (21).

### Definition

The definition of intensive statin treatment was atorvastatin 40 mg or rosuvastatin 20 mg per day. Subjects were categorized into current smoker or non-current smoker. Current smoker was defined as smoking on admission/quitting smoking for <3 months on admission/any cigarette use during follow-ups. The rest are defined as non-current smokers.

### Statistical Analyses

Normally distributed continuous variable was shown as the mean ± SD and compared by one-way analysis of variance (ANOVA). Non-normally distributed continuous variable was shown as median (interquartile range) and compared by the Kruskal-Wallis test. The categorical variable was represented as counts (proportions) and compared using the Chi-square test or Fisher's exact test (if the expected cell value was <5). *P*-value for trend (*P* trend) was calculated with a Wilcoxon type test for continuous variables or a linear-by-linear association for categorical variables across ordered HbA1c categories. The smooth curves visualized the association of HbA1c levels with vulnerability features using the locally weighted scatterplot smoothing (LOWESS) algorithm. The multivariable linear regression model was used to estimate the influence of HbA1c on the vulnerability features and lipid variability after adjusting various covariates involving demographic data, laboratory testing, and medications. Exploratory analyses were conducted in subgroups according to diabetes (yes or no), HbA1c categories (<5.7%, 5.7–6.4%, ≥6.5%), clinical presentation [acute coronary syndrome (ACS) or stable angina pectoris (SAP)], types of statins (atorvastatin or rosuvastatin), and lipid-lowering regimen (regular statins/intensive statins/statins plus ezetimibe).

A two-tailed *P*-value <0.05 was considered significant. Statistical analysis was performed using SPSS software version 18.0 (SPSS Inc., Chicago, IL, USA) and R version 3.5.1 (The R Foundation for Statistical Computing, Vienna, Austria).

## Result

### Patient Characteristics

Among the subjects who underwent elective PCI, 366 patients (58.2% ACS) were enrolled in the vulnerability feature analysis, and 4,445 patients (23.1% ACS) were enrolled in the lipid variability analysis. Patient characteristics have been summarized in Table 1 and Supplementary Tables S1, S2. Patients who received OCT examination were 61.4 ± 11.1 years, with 31.4% diabetes, 69.1% hypertension, and 52.5% dyslipidemia. The pre-procedure HbA1c level was 5.9 ± 1.3%. Among them, 60 (16.4%) subjects had a history of MI, and 79 (21.6%) subjects had a history of PCI. Pre-procedure pharmacologic therapies indicated that 34.4% of patients had statins more than 8 weeks, 35.2% had aspirin, and 27.3% had P2Y_12_ inhibitors. For patients who underwent scheduled follow-ups in lipid variability analysis, the average age was 63.8 ± 10.3 years, of which 64.0% had hypertension and 25.6% had diabetes. The average follow-up HbA1c level was 6.0 ± 1.2%.

**Table 1:** Patient characteristics.

	**Vulnerability analysis**	**Variability analysis**
	**(*n* = 366)**	**(*n* = 4,445)**
**Patient characteristics**		
Age, years	61.4 ± 11.1	63.8 ± 10.3
Male, *n* (%)	303 (82.8)	3,187 (71.7)
Current smoker, *n* (%)	144 (39.3)	1,001 (22.5)
Hypertension, *n* (%)	253 (69.1)	2,845 (64.0)
Diabetes mellitus, *n* (%)	115 (31.4)	1,139 (25.6)
Dyslipidemia, *n* (%)	192 (52.5)	2,148 (48.3)
Prior MI, *n* (%)	60 (16.4)	166 (3.7)
Prior PCI, *n* (%)	79 (21.6)	345 (7.7)
Prior CABG, *n* (%)	5 (1.4)	26 (0.6)
Ejection fraction, %	59.7 ± 9.6	64.8 ± 10.1
**Clinical presentation**, ***n*** **(%)**		
Acute coronary syndromes	213 (58.2)	1,028 (23.1)
Stable angina pectoris	153 (41.8)	3,417 (76.9)
**Target imaging vessel**, ***n*** **(%)**		
LAD	176 (48.1)	2,275 (49.4)
LCX	60 (16.4)	738 (16.0)
RCA	130 (35.5)	1,595 (34.6)
**Laboratory testing**		
HbA1c, %	5.90 ± 1.31	5.98 ± 1.16
LDL-C, mmol/L	2.98 ± 0.94	2.38 ± 0.97
HDL-C, mmol/L	1.17 ± 0.29	1.04 ± 0.28
Triglyceride, mmol/L	1.43 ± 0.84	1.77 ± 1.38
Total cholesterol, mmol/L	4.81 ± 1.06	4.35 ± 1.24
eGFR, ml/min/1.73 m^2^	70.6 ± 23.7	85.0 ± 19.7

*Values are mean ± SD or n (%). Dyslipidemia is defined LDL-C <1.8 mmol/L. OCT, optical coherence tomography; MI, myocardial infarction; PCI, percutaneous coronary intervention; CABG, coronary artery bypass grafting; LAD, left anterior descending artery; LCX, left circumflex artery; LDL-C, low-density lipoprotein cholesterol; HDL-C, high-density lipoprotein cholesterol; RCA, right coronary artery; eGFR, estimated glomerular filtration rate*.

### Vulnerability Features of Culprit Vessels

OCT findings were summarized in Table 2 according to pre-procedure HbA1c levels (Tertile1: HbA1c <5.7%, Tertile2: HbA1c 5.7–6.4%, Tertile3: HbA1c ≥6.5%). The vulnerability features were estimated by the minimal FCT, lipid index, macrophage index, and calcium index of the entire culprit vessel. Three-group comparisons identified a significant difference for minimal FCT (*P* = 0.008) and the pairwise comparisons indicated that HbA1c ≥6.5% group had a thinner minimal FCT than HbA1c 5.7–6.4% group (87.7 ± 44.2 vs. 101.1 ± 43.0 μm, *P* = 0.032) and HbA1c <5.7% group (87.7 ± 44.2 vs. 105.0 ± 53.4 μm, *P* = 0.003). Trend analysis showed a decreasing trend in minimum FCT with increasing HbA1c (*P* trend = 0.003). Besides, there was a significant difference in lipid index between three groups (*P* = 0.004). HbA1c ≥6.5% group showed a greater lipid index than HbA1c 5.7–6.4% group [1863.1 (1093.6, 2832.5) vs. 1521.8 (900.3, 2200.7) mm°, *P* = 0.046] and HbA1c <5.7% group [1863.1 (1093.6, 2832.5) vs. 1476.8 (847.7, 2213.7) mm°, *P* = 0.003], respectively. Trend analysis showed an increasing trend of lipid index with increasing HbA1c (*P* trend = 0.002). For macrophage and calcium feature assessment, HbA1c ≥6.5% group had a higher macrophage index [437.7 (291.1, 781.1) vs. 385.9 (194.8, 617.5) mm°, *P* = 0.037] and a greater calcium index [418.7 (100.6, 986.0) vs. 301.4 (129.2, 778.2) mm°, *P* = 0.023] than HbA1c <5.7% group. Trend analyses suggested that elevated HbA1c increased macrophage index (*P* trend = 0.049) and calcium index (*P* trend = 0.017). Compared to HbA1c <6.5 group, HbA1c ≥6.5 group had greater lesion length [24.00 (18.85, 29.50) vs. 22.20 (17.70, 27.35) mm, *P* = 0.019], higher prevalence of thrombus (56.6 vs. 42.4%, *P* = 0.024) and TCFA (50.6 vs. 37.5%, *P* = 0.041) (Supplementary Figure S3).

**Table 2:** Vulnerability features of the culprit vessel by OCT assessment according to HbA1c levels.

	**HbA1c <5.7%**	**HbA1c 5.7–6.4%**	**HbA1c ≥6.5%**	***P* value**	***P* trend**	**Pairwise comparison**		
	**(*n* = 191)**	**(*n* = 92)**	**(*n* = 83)**			***P*_1_**	***P*_**2**_**	***P*_3_**
Minimum lumen area, mm^2^	1.16 ± 0.92	1.15 ± 0.83	0.98 ± 0.61	0.378	0.150	–	0.118	–
Mean reference lumen area, mm^2^	7.8 ± 4.1	7.0 ± 2.3	6.3 ± 2.1	0.102	0.033*	–	0.038*	–
Percent area stenosis, %	81.7 ± 10.7	80.4 ± 12.6	82.5 ± 9.6	0.733	0.916	–	0.032*	–
Lesion length, mm	23.1 ± 8.0	23.2 ± 9.3	25.1 ± 8.6	0.190	0.113	–	0.082*	0.136
Plaque rupture, *n* (%)	79 (41.3)	27 (29.3)	30 (36.1)	0.143	0.239	0.033*	–	–
Thrombus, *n* (%)	90 (47.1)	30 (32.6)	47 (56.6)	0.005*	0.419	0.014*	0.094	0.001*
Thrombus with plaque rupture, *n* (%)	55 (28.8)	17 (18.5)	25 (30.1)	0.128	0.841	0.062	–	0.072
Thrombus without plaque rupture, *n* (%)	35 (18.3)	13 (14.1)	22 (26.5)	0.106	0.212	–	0.087	0.032*
Calcified nodule, *n* (%)	3 (2.2)	2 (2.9)	3 (4.6)	0.637	0.353	–	–	–
Microchannel, *n* (%)	71 (37.0)	36 (39.1)	37 (44.6)	0.513	0.265	–	0.154	–
Cholesterol crystal, *n* (%)	48 (25.1)	24 (26.1)	29 (34.9)	0.232	0.121	–	0.066	0.134*
Thin–cap fibroatheroma, *n* (%)	80 (41.8)	26 (28.2)	42 (50.6)	0.009*	0.458	0.018*	0.115	0.002*
Minimal fibrous cap thickness, μm	105.0 ± 53.4	101.1 ± 43.0	87.7 ± 44.2	0.008*	0.003*	–	0.003*	0.032*
**Lipid characteristics**								
Lipid index, degree × mm	1476.8 [847.7, 2213.7]	1521.8 [900.3, 2200.7]	1863.1 [1093.6, 2832.5]	0.004*	0.002*	–	0.003*	0.046*
Lipid length, mm	10.0 [7.0, 15.0]	11.0 [7.0, 14.0]	12.0 [8.0, 18.0]	0.020*	0.009*	–	0.017*	–
Max lipid angle, degree	238.8 [175.1, 305.8]	237.2 [183.4, 289.7]	249.5 [205.4, 309.5]	0.269	0.120	–	0.106	–
**Macrophage characteristics**								
Macrophage index, degree × mm	385.9 [194.8, 617.5]	412.9 [237.7, 727.3]	437.7 [291.1, 781.1]	0.067	0.049*	–	0.037*	–
Macrophage length, mm	7.0 [4.0, 11.0]	8.0 [4.0, 12.0]	9.0 [6.0, 13.0]	0.075	0.039*	–	0.041*	–
Max macrophage angle, degree	89.0 [66.6, 130.2]	97.5 [65.8, 134.7]	103.6 [69.7, 132.8]	0.412	0.186	–	–	–
**Calcium characteristics**								
Calcium index, degree × mm	301.4 [129.2, 778.2]	390.2 [182.3, 1114.0]	418.7 [100.6, 986.0]	0.051	0.017*	–	0.023*	–
Calcium length, mm	5.0 [2.0, 10.0]	6.0 [2.0, 12.0]	6.0 [2.0, 13.0]	0.274	0.110	–	–	–
Max calcium angle, degree	93.2 [63.9, 135.5]	119.8 [65.1, 190.0]	107.5 [65.8, 171.7]	0.115	0.128	–	–	–

Values are mean ± SD or median [interquartile range] for continuous variables and n (%) for categorical variables. P-value <0.2 for pairwise comparison was presented. Pairwise comparison P_1_ indicates P-value for HbA1c <5.7% vs. HbA1c 5.7–6.4%; P_2_, P value for HbA1c <5.7% vs. HbA1c ≥ 6.5%; P_3_, P value for HbA1c 5.7–6.4% vs. HbA1c ≥ 6.5%. *P <0.05.

### Effects of Pre-procedure HbA1c Levels on Vulnerability Features

**Figure 3:**
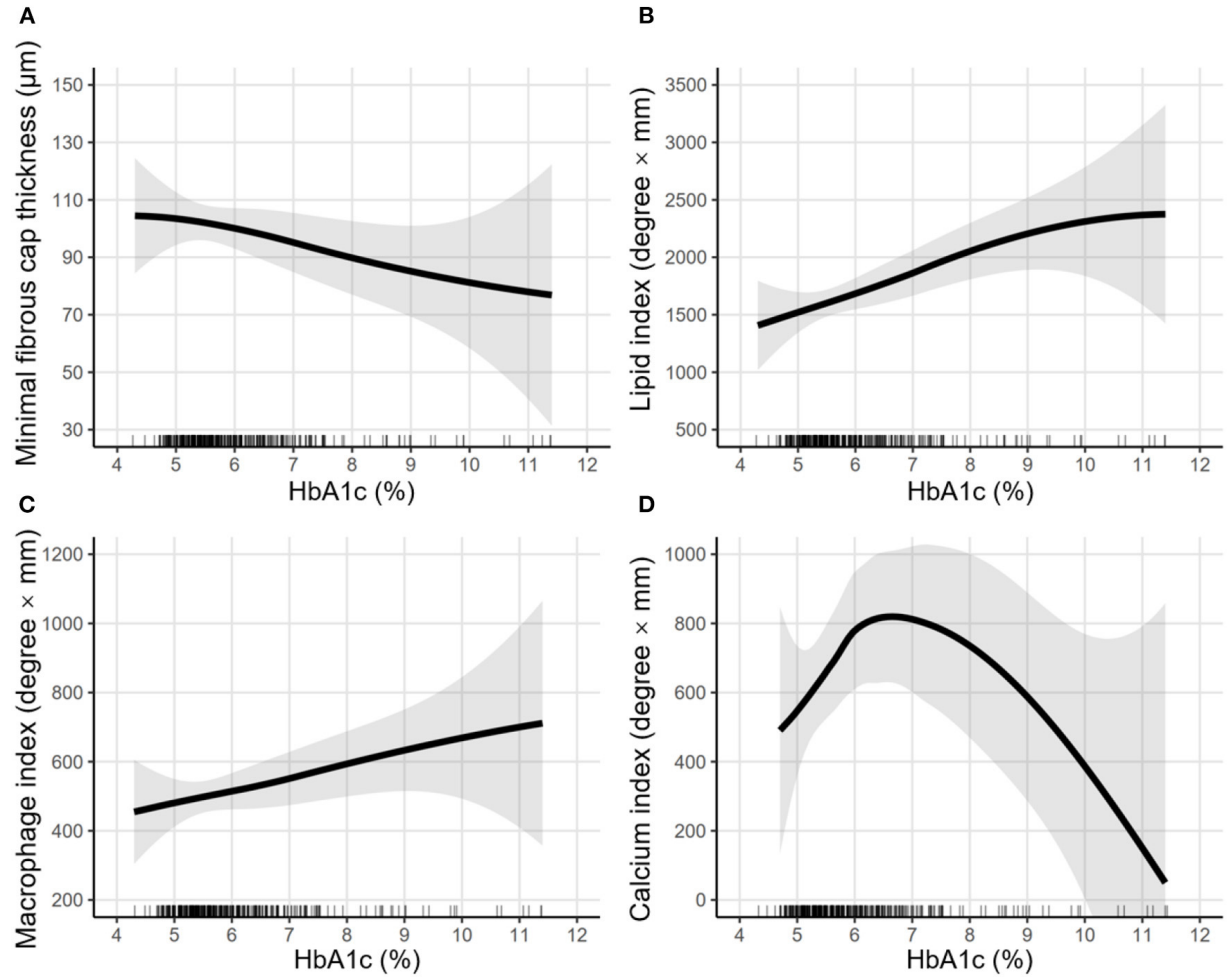
LOWESS curves of the association between pre-procedural HbA1c levels and vulnerability features. Locally weighted scatterplot smoothing (LOWESS) curves were used to visualize the rough association between pre-procedural HbA1c levels and vulnerability features, including **(A)** minimal fibrous cap thickness, **(B)** lipid index, **(C)** macrophage index, and **(D)** calcium index. The semi-transparent ribbon around the solid line indicates the 95% confidence interval. Rug plots show the distribution of pre-procedural HbA1c levels.

**Figure 4:**
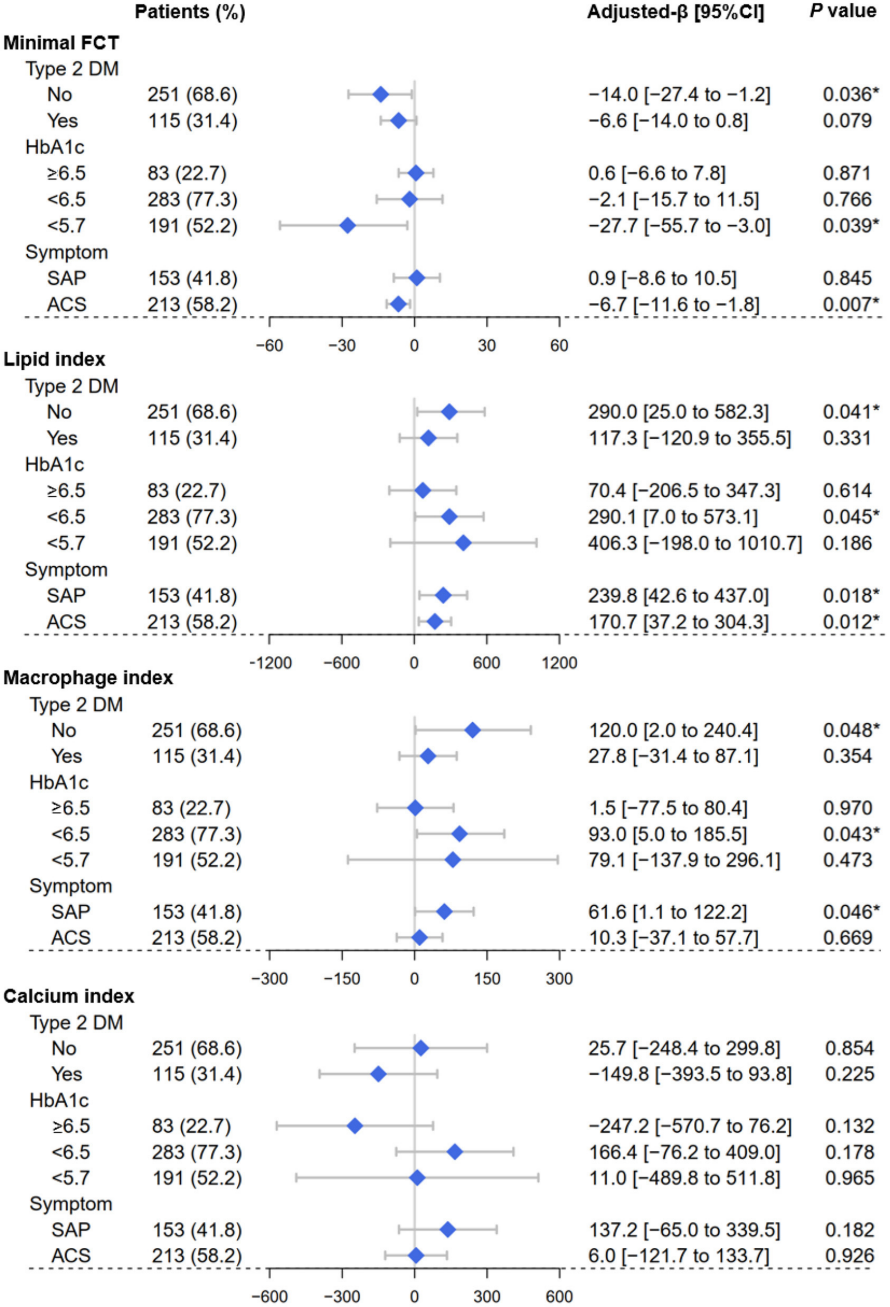
Forest plots of the vulnerability feature analyses. By using OCT assessment, forest plots depicted the effect of pre-procedure HbA1c levels on the vulnerability feature of culprit vessels, including minimal fibrous cap thickness, lipid index, macrophage index, and calcium index. Subgroups were determined according to Type 2 DM (yes or no), HbA1c categories (<5.7%, 5.7–6.4%, ≥6.5%), clinical symptom (SAP or ACS). OCT, optical coherence tomography; SAP, stable angina pectoris; ACS, acute coronary syndrome; FCT, fibrous cap thickness; DM, diabetes mellitus. *P < 0.05.

In Figure 3, LOWESS curves visualized the rough association of pre-procedural HbA1c levels with minimal FCT (downtrend), lipid index (uptrend), macrophage index (uptrend), and calcium index (reverse U-shaped). Linear regression models with multiple adjustments were subsequently performed and proved that elevated HbA1c was an independent risk factor for thinner minimal FCT [β = −6.985, 95% CI (−13.902 to −0.068), *P* = 0.048], higher lipid index [β = 226.299, 95% CI (67.977–384.621), *P* = 0.005], and greater macrophage index [β = 54.526, 95% CI (1.268–107.785), *P* = 0.045] (Table 3). Consistently, in non-diabetic CAD patients, elevated pre-procedure HbA1c still linearly decrease minimal FCT [β = −14.011, 95% CI (−27.393 to −1.221), *P* = 0.036], increase lipid index [β = 290.048, 95% CI (25.041–582.264), *P* = 0.041], and increase macrophage index [β = 120.029, 95% CI (2.031–240.362), *P* = 0.048] (Figure 4).

**Table 3:** Linear regression analyses of pre-procedure HbA1c levels on vulnerability features.

	**Model 1**		**Model 2**		**Model 3**	
	**Unadjusted-β [95% CI]**	***P*-value**	**Adjusted-β [95% CI]**	***P*-value**	**Adjusted-β [95% CI]**	***P*-value**
Minimal FCT	−4.528 [−9.308 to 0.252]	0.063	−6.735 [−13.589 to 0.119]	0.054	−6.985 [−13.902 to −0.068]	0.048*
Lipid index	240.686 [133.924 to 347.448]	<0.001*	226.835 [71.11 to 382.561]	0.004*	226.299 [67.977 to 384.621]	0.005*
Macrophage index	38.248 [2.206 to 74.29]	0.038*	57.451 [5.227 to 109.675]	0.031*	54.526 [1.268 to 107.785]	0.045*
Calcium index	31.816 [−75.623 to 139.255]	0.560	−100.204 [−255.258 to 54.849]	0.204	−81.223 [−239.805 to 77.358]	0.314

*Model 1 adjusted for none.*

*Model 2 adjusted for age, male, diabetes, hypertension, prior myocardial infarction, prior percutaneous coronary intervention, current smoker, low-density lipoprotein cholesterol, ejection fraction, estimated glomerular filtration rate.*

*Model 3 additionally adjusted for covariates of pre-procedure medications, including statin, aspirin, P2Y_12_ inhibitor, insulin treatment.*

FCT indicates fibrous cap thickness; CI, confidence interval. *P <0.05.

### The Visit-to-Visit Variability of Lipid Profile During Follow-Ups

Patient characteristics of variability analysis were summarized in Supplementary Table S2 according to the average follow-up HbA1c categories (Tertile1: HbA1c <5.7%, Tertile2: HbA1c 5.7–6.4%, Tertile3: HbA1c ≥6.5%). Three-group comparisons indicated that elevated follow-up average HbA1c levels were associated with greater VIM of lipid profiles, including LDL-C (Tertile1: 74.8 ± 44.2; Tertile2: 76.0 ± 47.5; Tertile3: 80.0 ± 46.9/1,000, *P* = 0.029), HDL-C (Tertile1: 30.5 ± 12.6; Tertile2: 30.7 ± 13.9; Tertile3: 32.9 ± 15.4/1,000, *P* < 0.001), non-HDL-C (Tertile1: 20.0 ± 14.9; Tertile2: 20.9 ± 17.1; Tertile3: 22.8 ±1 8.0/1,000, *P* = 0.001), TC (Tertile1: 7.7 ± 6.5; Tertile2: 8.4 ± 7.3; Tertile3: 9.7 ± 8.3/1,000, *P* < 0.001), and TG (Tertile1: 27.6 ± 27.9; Tertile2: 27.3 ± 25.5; Tertile3: 35.4 ± 40.7/1,000, *P* < 0.001) (Supplementary Table S2). The consistent results were confirmed when SD or CV was used to estimate variability (Supplementary Table S2).

### Effects of Follow-Up HbA1c Levels on Lipid Variability

Multivariable linear regression analyses were performed and found that elevated follow-up HbA1c led to greater VIM of lipid profiles, including LDL-C [β = 2.594, 95% CI (1.175–4.013), *P* < 0.001], HDL-C [β = 0.461, 95% CI (0.012–0.911), *P* = 0.044], Non-HDL-C [β = 1.473, 95% CI (0.926–2.021), *P* < 0.001], TC [β = 0.947, 95% CI (0.721–1.174), *P* < 0.001], and TG [β = 4.217, 95% CI (3.186–5.249), *P* < 0.001] (Table 4). Consistently, the findings remained when SD or CV was employed (Supplementary Tables S4, S5).

**Table 4:** Linear regression analyses of average follow-up HbA1c levels on the visit-to-visit variability of lipid profiles.

**The VIM of** **lipid profiles**	**Model 1**		**Model 2**		**Model 3**	
	**Unadjusted-β [95% CI]**	***P*-value**	**Adjusted-β [95% CI]**	***P*-value**	**Adjusted-β [95% CI]**	***P*-value**
LDL-C	2.802 [1.600–4.005]	<0.001	3.465 [1.953–4.978]	<0.001	2.594 [1.175–4.013]	<0.001
HDL-C	0.634 [0.271–0.996]	0.001	0.544 [0.090–0.998]	0.019	0.461 [0.012–0.911]	0.044
Non-HDL-C	1.221 [0.777–1.666]	<0.001	1.653 [1.094–2.213]	<0.001	1.473 [0.926–2.021]	<0.001
TC	0.849 [0.659–1.039]	<0.001	1.069 [0.831–1.308]	<0.001	0.947 [0.721–1.174]	<0.001
TG	3.710 [2.895–4.526]	<0.001	4.345 [3.321–5.370]	<0.001	4.217 [3.186–5.249]	<0.001

*Model 1 adjusted for none.*

*Model 2 adjusted for age, male, diabetes, hypertension, prior myocardial infarction, prior percutaneous coronary intervention, current smoker, ejection fraction, estimated glomerular filtration rate.*

*Model 3 additionally adjusted for covariates of medications during follow-up, including the type of statin (atorvastatin/rosuvastatin/others), the intensive statin treatment (vs. regular), statin combined with ezetimibe treatment (vs. without), insulin treatment (vs. without).*

*Lipid variability was represented by VIM. VIM, variability independent of the mean; LDL-C, low-density lipoprotein cholesterol; HDL-C, high-density lipoprotein cholesterol; TC, total cholesterol; TG, triglyceride; CI, confidence interval*.

**Figure 5:**
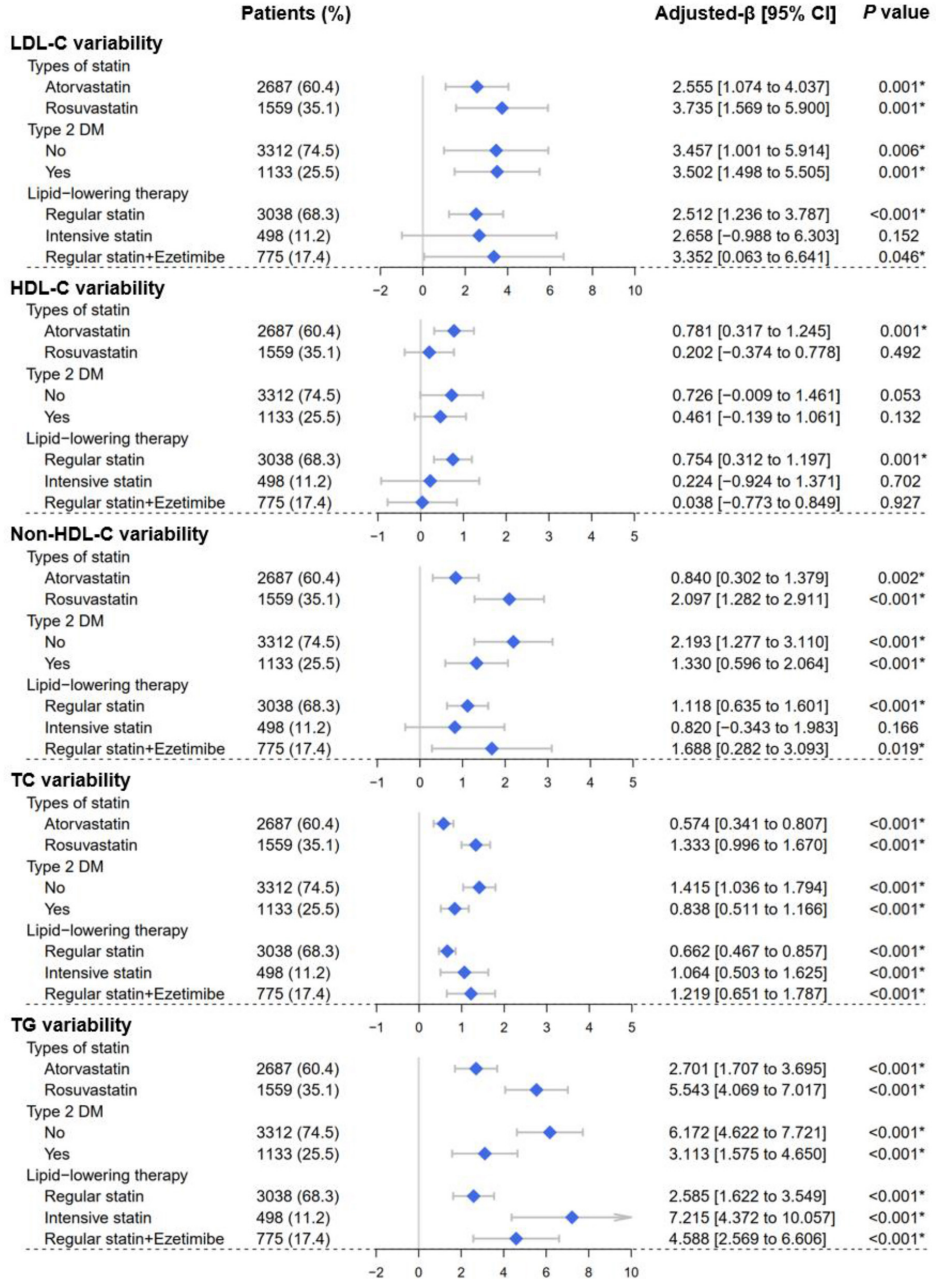
Forest plots of the lipid variability analyses. Forest plots depicted the effect of average follow-up HbA1c levels on visit-to-visit variability of lipid profiles, including LDL-C, HDL-C, Non-HDL-C, TC, and TG. Lipid variability was represented by the variability independent of the mean (VIM). Subgroups were determined according to Type 2 DM (yes or no), types of statins (atorvastatin or rosuvastatin), and lipid-lowering therapy strategy (regular statins/intensive statins/statins plus ezetimibe). LDL-C, low-density lipoprotein cholesterol; HDL-C, high-density lipoprotein cholesterol; Non-HDL-C, non-high-density lipoprotein cholesterol; TC, total cholesterol; TG, triglyceride; DM, diabetes mellitus. *P < 0.05.

Consistently, in non-diabetic patients, elevated follow-up HbA1c levels still increased the VIM of lipid profiles, including LDL-C [β = 3.457, 95% CI (1.001–5.914), *P* = 0.006], Non-HDL-C [β = 2.193, 95% CI (1.277–3.110), *P* < 0.001], TC (β = 1.415, 95% CI (1.036–1.794), *P* < 0.001], and TG [β = 6.172, 95% CI (4.622–7.721), *P* < 0.001] (Figure 5). Consistently, the findings remained when SD or CV was employed (Supplementary Figures S1, S2).

## Discussion

This retrospective observational study enrolled the patient who underwent elective PCI. By using OCT assessment, elevated pre-procedure HbA1c level was verified to increase the plaque vulnerability of culprit vessels, including thinner minimal FCT, higher lipid index, and greater macrophage index. Besides, elevated average follow-up HbA1c level was identified as an independent risk factor for higher visit-to-visit variability of lipids, including LDL-C, HDL-C, non-HDL-C, TC, and TG. Exploratory analyses also confirmed that the above findings were consistent in non-diabetic patients.

Due to the high resolution (10–15 μm), OCT provides a detailed depiction of the vulnerable features in atherosclerotic lesions, which has been recognized as the gold standard for coronary morphology evaluation (22). Numerous studies have shown that abnormal glucose metabolism is associated with increased plaque vulnerability, which thus leads to the incidence of adverse cardiovascular events (2). By using OCT assessment, Milzi et al. found that the presence of type 2 DM was associated with a thinner FCT in CAD patients (23). Suzuki et al. found that patients with impaired glucose tolerance had larger lipid cores and thinner FCT compared to patients with normal glucose tolerance (24). Kato et al. proved that macrophage infiltration was more frequent in patients with HbA1c ≥8% (25). By using magnetic resonance imaging, Sun et al. found that elevated HbA1c was correlated with greater carotid plaque vulnerability (26). Consistently, by using OCT assessment in 366 independent CAD patients, the current study demonstrated that elevated HbA1c increased the atherosclerotic plaque vulnerability, including thinner minimal FCT, greater lipid index, and higher macrophage index.

Some underlying mechanisms may be involved in the vulnerable features. For features of lipid and FCT, the elevation of HbA1c may contribute to lipid accumulation in culprit vessels by directly raising serum atherogenic lipid levels. In previous studies, increased glucose levels have been verified to up-regulated atherogenic lipid levels throughout the entire range of blood glucose (27, 28). Elevated atherogenic lipids can lead to lipid accumulation in the coronary artery, which thus promotes the progression of atherosclerosis and makes FCT thinner (29).

For the macrophage features, elevated HbA1c may increase macrophage infiltration by up-regulating the level of chronic inflammation. Chronic exposure to hyperglycemia and insulin resistance has been found to up-regulate inflammation levels through endoplasmic reticulum stress and mitochondrial superoxide overproduction, thereby promoting macrophage adhesion to the vascular wall and the development of atherosclerosis (30, 31). Indeed, the up-regulation of inflammation levels has been detected as early as pre-diabetes status (32).

For calcium features, it is still a controversial issue regarding the association between abnormal glucose metabolism and coronary calcium deposits. Some studies have shown that the presence of type 2 DM increases the calcium burden in the coronary artery (33, 34). While by using OCT assessment, Milzi et al. proved that the presence of type 2 DM was not related to calcium deposits in culprit lesions (23). In the current study, the pairwise comparison indicated that HbA1c ≥6.5% group had a greater calcium index than HbA1c <5.7% group [418.7 (100.6, 986.0) vs. 301.4 (129.2, 778.2) mm°, *P* = 0.023]. However, no significant linear correlation was found between HbA1c and calcium index. This may be due to the fact that calcium index is less affected by HbA1c levels, compared to other vulnerable features.

The visit-to-visit variability of lipid profiles has been identified as an independent risk factor for adverse cardiovascular events (17). By intravascular ultrasound examination, the association between higher lipid variability and greater plaque vulnerability has been revealed (12). Furthermore, the current study verified the close relationship between the elevated follow-up HbA1c levels and the visit-to-visit variability of lipid profiles, which was also confirmed in non-diabetic patients.

Although the mechanism is not entirely clear, several possible explanations are worth considering. On the one hand, genome-wide association analysis has indicated an underlying association between glucose dysregulation of lipid variability. For instance, DKK3 (Dickkopf-3) gene expression is positively correlated to the visit-to-visit variability of HDL-C (19). Meanwhile, DKK3 was aberrantly expressed in β-cells of patients with type 2 diabetes, which can inhibit the Wnt signaling pathway, thereby depressing the survival and proliferation of β cells (20). The suppression of β cells could lead to an increased HbA1c level. On the other hand, medication non-compliance is also one of the potential explanations. Medication non-compliance has been shown to not only increase the visit-to-visit lipid variability but is also related to poor glycemic control in diabetic patients (35, 36). Therefore, the positive correlation between HbA1c levels and lipid variability may partly result from medication non-compliance.

Some new insights in the current study are worth noting. On the one hand, the current study confirmed that even in non-diabetic patients, elevated HbA1c increases not only plaque vulnerability but also the visit-to-visit lipid variability. These results provide a novel idea for preventive medicine that preventive glucose management may benefit non-diabetic CAD patients. On the other hand, the current study verified the association of elevated HbA1c with higher lipid index and greater macrophage index in patients with SAP, who had a relatively fewer plaques rupture event. This indicates that elevated HbA1c levels have already exerted an influence on the plaque vulnerability before plaque rupture.

The current study still has several limitations. First, the inherent bias cannot be eliminated due to the retrospective design. Second, HbA1c levels can be affected by antidiabetic therapy. The regression analysis has adjusted for the covariate of insulin therapy. However, detailed antidiabetic therapy was not further adjusted and may introduce potential bias. Third, some patients did not strictly comply with the follow-up procedures scheduled at 1st, 3rd, 6th, 9th, and 12th months following PCI, which may affect the assessment of lipid variability. Finally, the current study did not address the long-term clinical outcome, which was expected in further studies.

## Conclusion

In patients undergoing elective PCI, elevated HbA1c increases the atherosclerotic plaque vulnerability and the visit-to-visit variability of lipid profiles, which is consistent in non-diabetic patients.

## Data Availability Statement

The raw data supporting the conclusions of this article will be made available by the authors, without undue reservation.

## Ethics Statement

The studies involving human participants were reviewed and approved by the Ethics Committee of Sir Run Run Shaw Hospital of Zhejiang University. Written informed consent for participation was not required for this study in accordance with the national legislation and the institutional requirements.

## Author Contributions

WZ, ZC, and XS conceived and designed the study. DL organized these data and drafted the manuscript with the help of YLi, CW, and HJ. DL analyzed the data. LZ drew the pictures. XH, ML, and YLu detected any errors in the whole process. All authors have read and approved the manuscript for submission.

## Funding

This work was supported by grants from the National Natural Science Foundation of China (82070408), the Medical Health Science and Technology Project of Zhejiang Provincial Health Commission (2021RC014), the Traditional Chinese Medicine Science and Technology Project of Zhejiang Province (2021ZB172), and the Joint Funds for the Innovation of Science and Technology, Fujian province (2018Y9094).

## Conflict of Interest

The authors declare that the research was conducted in the absence of any commercial or financial relationships that could be construed as a potential conflict of interest. The reviewer HM declared a shared affiliation, though no other collaboration, with several of the authors, HJ and LZ, to the handling editor.

## Publisher's Note

All claims expressed in this article are solely those of the authors and do not necessarily represent those of their affiliated organizations, or those of the publisher, the editors and the reviewers. Any product that may be evaluated in this article, or claim that may be made by its manufacturer, is not guaranteed or endorsed by the publisher.

## Abbreviations

CAD: coronary artery disease
DM: diabetes mellitus
HbA1c: hemoglobin A1c
ADA: the American Diabetes Association
WHO: the World Health Organization
MI: myocardial infarction
PCI: percutaneous coronary intervention
OCT: optical coherence tomography
FCT: fibrous cap thickness
LDL-C: low-density lipoprotein cholesterol
HDL-C: high-density lipoprotein cholesterol
non-HDL-C: non-high-density lipoprotein cholesterol
TC: total cholesterol
TG: triglyceride
TIMI: Thrombolysis in Myocardial Infarction
ICC: intraclass correlation coefficient
SD: standard deviation
CV: coefficient of variation
VIM: variability independent of the mean
eGFR: estimated glomerular filtration rate
LOWESS: locally weighted scatterplot smoothing
ACS: acute coronary syndromes
SAP: stable angina pectoris.
