# Supplementary material for: Elevation of Hemoglobin A1c Increases the Atherosclerotic Plaque Vulnerability and the Visit-to-Visit Variability of Lipid Profiles in Patients Who Underwent Elective Percutaneous Coronary Intervention
Source: Front Cardiovasc Med. 2022 Feb 3;9:803036. doi: 10.3389/fcvm.2022.803036 (PMC8852677; doi:10.3389/fcvm.2022.803036)
Supplement: Supplementary file 1 [file Data_Sheet_1.docx]

**Supplementary materials**

**Supplementary tables**

**Table S1.** Patient characteristics of plaque vulnerability analysis

**Table S2.** Patient characteristics of lipid variability analysis

**Table S3.** OCT findings of lesion severity in the overall population

**Table S4.** Linear regression analysis of the follow-up HbA1c on the CV of lipid profiles

**Table S5.** Linear regression analysis of the follow-up HbA1c on the SD of lipid profiles

**Supplementary figures**

**Figure S1.** Forest plot for lipid variability represented by CV

**Figure S2.** Forest plot for lipid variability represented by SD

**Table S1.** Patient characteristics of plaque vulnerability analysis

|  | Overall | HbA1c <5.7% | HbA1c 5.7-6.4% | HbA1c ≥6.5% | *P* value |
| --- | --- | --- | --- | --- | --- |
|  | (n=366) | (n=191) | (n=92) | (n=83) |  |
| Patient characteristics |  |  |  |  |  |
| Age, years | 61.4±11.1 | 62.4±11.2 | 61.6±10.4 | 58.8±11.2 | 0.044* |
| Male, n (%) | 303 (82.8) | 161 (84.3) | 74 (80.4) | 68 (81.9) | 0.703 |
| Current smoker, n (%) | 144 (39.3) | 77 (40.3) | 35 (38.0) | 32 (38.6) | 0.922 |
| Dyslipidemia, n (%) | 192 (52.5) | 85 (44.5) | 53 (57.6) | 54 (65.1) | 0.004* |
| Hypertension, n (%) | 253 (69.1) | 127 (66.5) | 68 (73.9) | 58 (69.9) | 0.442 |
| Prior MI, n (%) | 60 (16.4) | 26 (13.6) | 21 (22.8) | 13 (15.7) | 0.143 |
| Prior PCI, n (%) | 79 (21.6) | 35 (18.3) | 25 (27.2) | 19 (22.9) | 0.225 |
| Prior CABG, n (%) | 5 (1.4) | 2 (1.0) | 2 (2.2) | 1 (1.2) | 0.739 |
| Ejection fraction, % | 59.7±9.6 | 60.4±9.6 | 59.0±9.5 | 58.8±9.5 | 0.352 |
| Clinical presentation, n (%) |  |  |  |  | 0.029* |
| Acute coronary syndromes | 213 (58.2) | 121 (63.4) | 43 (46.7) | 49 (59.0) |  |
| Stable coronary artery disease | 153 (41.8) | 70 (36.6) | 49 (53.3) | 34 (41.0) |  |
| Target imaging vessel, n (%) |  |  |  |  | 0.277 |
| LAD | 176 (48.1) | 83 (43.5) | 53 (57.6) | 40 (48.2) |  |
| LCX | 60 (16.4) | 33 (17.3) | 13 (14.1) | 14 (16.9) |  |
| RCA | 130 (35.5) | 75 (39.3) | 26 (28.3) | 29 (34.9) |  |
| Laboratory data |  |  |  |  |  |
| LDL-C, mmol/L | 2.98±0.94 | 2.99±0.90 | 3.03±0.97 | 2.92±1.00 | 0.760 |
| HDL-C, mmol/L | 1.17±0.29 | 1.22±0.31 | 1.11±0.25 | 1.12±0.27 | 0.002* |
| Triglyceride, mmol/L | 1.43±0.84 | 1.3±0.66 | 1.47±0.74 | 1.67±1.17 | 0.002* |
| Total cholesterol, mmol/L | 4.81±1.06 | 4.83±1.03 | 4.83±1.11 | 4.75±1.09 | 0.819 |
| eGFR, ml/min/1.73m^2^ | 70.6±23.7 | 70.5±23.9 | 69.5±21.1 | 72.0±25.4 | 0.603 |
| Pre-admission medication, n (%) |  |  |  |  |  |
| Aspirin | 129 (35.2) | 63 (33.0) | 41 (44.6) | 25 (30.1) | 0.087 |
| P2Y_12_ inhibitor | 100 (27.3) | 45 (23.6) | 32 (34.8) | 23 (27.7) | 0.139 |
| Statin | 126 (34.4) | 54 (28.3) | 38 (41.3) | 34 (41.0) | 0.035 |
| Insulin | 13 (3.6) | 1 (0.5) | 2 (2.2) | 10 (12.0) | <0.001* |

Values are mean ± SD or n (%). Dyslipidemia is defined as LDL-C less than 1.8 mmol/L. OCT indicates optical coherence tomography; MI, [myocardial](javascript:;) [infarction](javascript:;); PCI, percutaneous coronary intervention; CABG, coronary artery bypass grafting; LAD, left anterior descending artery; LCX, left circumflex artery; RCA, right coronary artery; LDL-C, low-density lipoprotein cholesterol; HDL-C, high-density lipoprotein cholesterol; eGFR, estimated glomerular filtration rate. **P* <0.05

**Table S2.** Patient characteristics of lipid variability analysis

|  | Overall | HbA1c <5.7% | HbA1c 5.7-6.4% | HbA1c ≥6.5% | *P* value |
| --- | --- | --- | --- | --- | --- |
|  | (n=4445) | (n=1536) | (n=1608) | (n=1301) |  |
| Patient characteristics |  |  |  |  |  |
| Age, years | 63.8±10.3 | 62.4±10.7 | 64.8±10 | 64.3±10.1 | <0.001* |
| Male, n (%) | 3187 (71.7) | 1155 (75.2) | 1122 (69.8) | 910 (69.9) | 0.005* |
| Current smoker, n (%) | 1001 (22.5) | 409 (26.6) | 312 (19.4) | 280 (21.5) | 0.001* |
| Hypertension, n (%) | 2845 (64.0) | 900 (58.6) | 1037 (64.5) | 908 (69.8) | 0.001* |
| Dyslipidemia, n (%) | 2148 (48.3) | 737 (48.0) | 793 (49.3) | 618 (47.5) | 0.684 |
| Prior MI, n (%) | 166 (3.7) | 49 (3.2) | 69 (4.3) | 48 (3.7) | 0.516 |
| Prior PCI, n (%) | 345 (7.7) | 111 (7.2) | 142 (8.8) | 92 (7.1) | 0.456 |
| Prior CABG, n (%) | 26 (0.6) | 0 (0) | 14 (0.9) | 12 (0.9) | <0.001* |
| Ejection fraction, % | 64.8 (10.1) | 65.6 (9.4) | 65.3 (11.1) | 63.0 (11.5) | 0.107 |
| eGFR, ml/min/1.73m^2^ | 85.0±19.7 | 87.5±17.5 | 83.3±19.0 | 84.0±22.4 | <0.001* |
| Clinical presentation, n (%) |  |  |  |  | 0.001* |
| Acute coronary syndromes | 1028 (23.1) | 310 (20.2) | 378 (23.5) | 340 (26.1) |  |
| Stable coronary artery disease | 3417 (76.9) | 1226 (79.8) | 1230 (76.5) | 961 (73.9) |  |
| Target imaging vessel, n (%) |  |  |  |  | <0.001* |
| RCA | 1595 (34.6) | 578 (37.6) | 529 (34.4) | 488 (31.8) |  |
| LAD | 2275 (49.4) | 740 (48.2) | 801 (52.1) | 734 (47.8) |  |
| LCX | 738 (16.0) | 218 (14.2) | 206 (13.4) | 314 (20.4) |  |
| Follow-up lipid profiles, mmol/L |  |  |  |  |  |
| LDL-C | 1.87±0.60 | 1.85±0.58 | 1.89±0.62 | 1.86±0.59 | 0.203 |
| HDL-C | 1.05±0.25 | 1.08±0.25 | 1.06±0.24 | 1.01±0.26 | <0.001* |
| Non-HDL-C | 2.57±0.73 | 2.54±0.68 | 2.61±0.76 | 2.58±0.76 | 0.091 |
| Total cholesterol | 3.75±0.80 | 3.73±0.73 | 3.79±0.82 | 3.74±0.84 | 0.160 |
| Triglyceride | 1.56±0.85 | 1.48±0.74 | 1.56±0.74 | 1.66±1.07 | <0.001* |
| SD, mmol/(1000×L) |  |  |  |  |  |
| LDL-C | 542.1±367.3 | 528.2±359.8 | 543.5±378.5 | 556.7±361.7 | 0.204 |
| HDL-C | 141.5±85.2 | 141.0±81.7 | 139.8±87.5 | 144.1±86.3 | 0.480 |
| Non-HDL-C | 522.4±381.2 | 495.8±336.8 | 523.0±388.3 | 553.1±418.4 | 0.004* |
| Total cholesterol | 689.7±464.4 | 646.8±426.5 | 686.8±467.3 | 744.0±497.4 | <0.001* |
| Triglyceride | 483.5±618.1 | 435.1±537.5 | 446.5±476.8 | 586.4±817.7 | <0.001* |
| CV, /1000 |  |  |  |  |  |
| LDL-C | 286.4±165.6 | 279.6±158.8 | 283.5±169.9 | 298.1±167.7 | 0.029* |
| HDL-C | 136.9±86.1 | 131.6±73.9 | 134.0±83.9 | 146.8±100.3 | <0.001* |
| Non-HDL-C | 199.1±122.9 | 191.6±112.1 | 196.8±125.1 | 210.7±131.5 | 0.002* |
| Total cholesterol | 180.4±106.8 | 169.3±98.0 | 177.8±106.1 | 196.5±115.5 | <0.001* |
| Triglyceride | 277.5±165.5 | 266.2±153.8 | 264.8±148.9 | 306.6±192.6 | <0.001* |
| VIM, /1000 |  |  |  |  |  |
| LDL-C | 76.7±46.3 | 74.8±44.2 | 76.0±47.5 | 80.0±46.9 | 0.029* |
| HDL-C | 31.3±13.9 | 30.5±12.6 | 30.7±13.9 | 32.9±15.4 | <0.001* |
| Non-HDL-C | 21.2±16.7 | 20.0±14.9 | 20.9±17.1 | 22.8±18.0 | 0.001* |
| Total cholesterol | 8.5±7.4 | 7.7±6.5 | 8.4±7.3 | 9.7±8.3 | <0.001* |
| Triglyceride | 29.8±31.6 | 27.6±27.9 | 27.3±25.5 | 35.4±40.7 | <0.001* |
| Follow-up medications, n (%) |  |  |  |  |  |
| Aspirin | 4295 (96.6) | 1485 (96.7) | 1557 (96.8) | 1253 (96.3) | 0.779 |
| P2Y_12_ Inhibitor | 3591 (80.8) | 1287 (83.8) | 1298 (80.7) | 1006 (77.3) | 0.001* |
| Statin | 4400 (99.0) | 1521 (99.0) | 1595 (99.2) | 1284 (98.7) | 0.491 |
| Intensive statin | 528 (11.9) | 190 (12.4) | 162 (10.1) | 176 (13.5) | 0.041* |
| Ezetimibe | 920 (20.7) | 295 (19.2) | 367 (22.8) | 258 (19.8) | 0.067 |

Values are mean ± SD or n (%). SD indicates standard deviation; CV, coefficient of variation; VIM, variability independent of the mean; other abbreviations, refer to Table S1. **P* <0.05.

**Table S3.** OCT findings of lesion severity in the overall population

|  | overall | HbA1c<6.5 | HbA1c≥6.5 | *P* value^†^ |
| --- | --- | --- | --- | --- |
| Lesion length, mm | 22.80 [17.95, 27.81] | 22.20 [17.70, 27.35] | 24.00 [18.85, 29.50] | 0.019* |
| Plaque rupture, n (%) | 136 (37.2) | 106 (37.5) | 30 (36.1) | 0.897 |
| Thrombus, n (%) | 167 (45.6) | 120 (42.4) | 47 (56.6) | 0.024* |
| Thrombus with plaque rupture, n (%) | 97 (26.5) | 72 (25.4) | 25 (30.1) | 0.399 |
| Thrombus without plaque rupture, n (%) | 70 (19.1) | 48 (17.0) | 22 (26.5) | 0.058 |
| Thin-cap fibroatheroma, n (%) | 148 (40.4) | 106 (37.5) | 42 (50.6) | 0.041* |

†Statistical differences were tested by Chi-square test or Fisher’s exact test for categorical variable and Kruskal-Wallis test for continuous variable.

**Table S4.** Linear regression analyses of follow-up HbA1c levels on the CV of lipid profiles

| The CV of  lipid profiles | Model 1 |  |  | Model 2 |  |  | Model 3 |  |
| --- | --- | --- | --- | --- | --- | --- | --- | --- |
|  | Unadjusted-β [95% CI] | *P* value |  | Adjusted-β [95% CI] | *P* value |  | Adjusted-β [95% CI] | *P* value |
| LDL-C | 10.060 [5.755 to 14.365] | <0.001 |  | 12.451 [7.034 to 17.867] | <0.001 |  | 9.33 [4.247 to 14.412] | <0.001 |
| HDL-C | 3.947 [1.706 to 6.189] | 0.001 |  | 3.579 [0.777 to 6.381] | 0.012 |  | 2.86 [0.113 to 5.607] | 0.041 |
| Non-HDL-C | 8.893 [5.612 to 12.173] | <0.001 |  | 12.087 [7.958 to 16.216] | <0.001 |  | 10.729 [6.689 to 14.769] | <0.001 |
| TC | 11.943 [9.187 to 14.699] | <0.001 |  | 15.002 [11.543 to 18.460] | <0.001 |  | 13.198 [9.915 to 16.481] | <0.001 |
| TG | 18.317 [14.046 to 22.588] | <0.001 |  | 20.982 [15.617 to 26.347] | <0.001 |  | 20.181 [14.785 to 25.577] | <0.001 |

Model 1 adjusted for none.

Model 2 adjusted for age, male, diabetes, hypertension, prior myocardial infarction, prior percutaneous coronary intervention, current smoker, ejection fraction, estimated glomerular filtration rate.

Model 3 additionally adjusted for covariates of treatment strategy during follow-ups, including the type of statin (atorvastatin/ rosuvastatin/ others), the intensive statin treatment (*vs.* regular), statin combined with ezetimibe treatment (*vs.* without), insulin treatment (*vs.* without).

CV indicates the coefficient of variation; LDL-C, low-density lipoprotein cholesterol; HDL-C, high-density lipoprotein cholesterol; TC, total cholesterol; TG, triglyceride; CI, confidence interval.

**Table S5.** Linear regression analyses of follow-up HbA1c levels on the SD of lipid profiles

|  | Model 1 |  |  | Model 2 |  |  | Model 3 |  |
| --- | --- | --- | --- | --- | --- | --- | --- | --- |
|  | Unadjusted-β [95% CI] | *P* value |  | Adjusted-β [95% CI] | *P* value |  | Adjusted-β [95% CI] | *P* value |
| LDL-C | 19.247 [9.692 to 28.802] | <0.001 |  | 19.469 [9.059 to 29.879] | <0.001 |  | 15.933 [6.061 to 25.805] | 0.002 |
| HDL-C | 0.645 [-1.577 to 2.866] | 0.569 |  | 2.489 [-0.177 to 5.155] | 0.067 |  | 2.453 [-0.219 to 5.125] | 0.072 |
| Non-HDL-C | 28.925 [18.755 to 39.094] | <0.001 |  | 28.599 [17.476 to 39.723] | <0.001 |  | 26.446 [15.509 to 37.383] | <0.001 |
| TC | 48.040 [36.041 to 60.038] | <0.001 |  | 50.995 [37.584 to 64.406] | <0.001 |  | 46.96 [34.092 to 59.828] | <0.001 |
| TG | 70.123 [54.183 to 86.064] | <0.001 |  | 32.397 [19.468 to 45.327] | <0.001 |  | 30.881 [17.849 to 43.914] | <0.001 |

Model 1 adjusted for none.

Model 2 adjusted for age, male, diabetes, hypertension, prior myocardial infarction, prior percutaneous coronary intervention, current smoker, ejection fraction, estimated glomerular filtration rate, the average level of corresponding lipid.

Model 3 additionally adjusted for covariates of treatment strategy during follow-ups, including the type of statin (atorvastatin/ rosuvastatin/ others), the intensive statin treatment (*vs.* regular), statin combined with ezetimibe treatment (*vs.* without), insulin treatment (*vs.* without).

SD indicates standard deviation; LDL-C, low-density lipoprotein cholesterol; HDL-C, high-density lipoprotein cholesterol; TC, total cholesterol; TG, triglyceride; CI, confidence interval.

**Figure S1.** Forest plot for lipid variability represented by CV


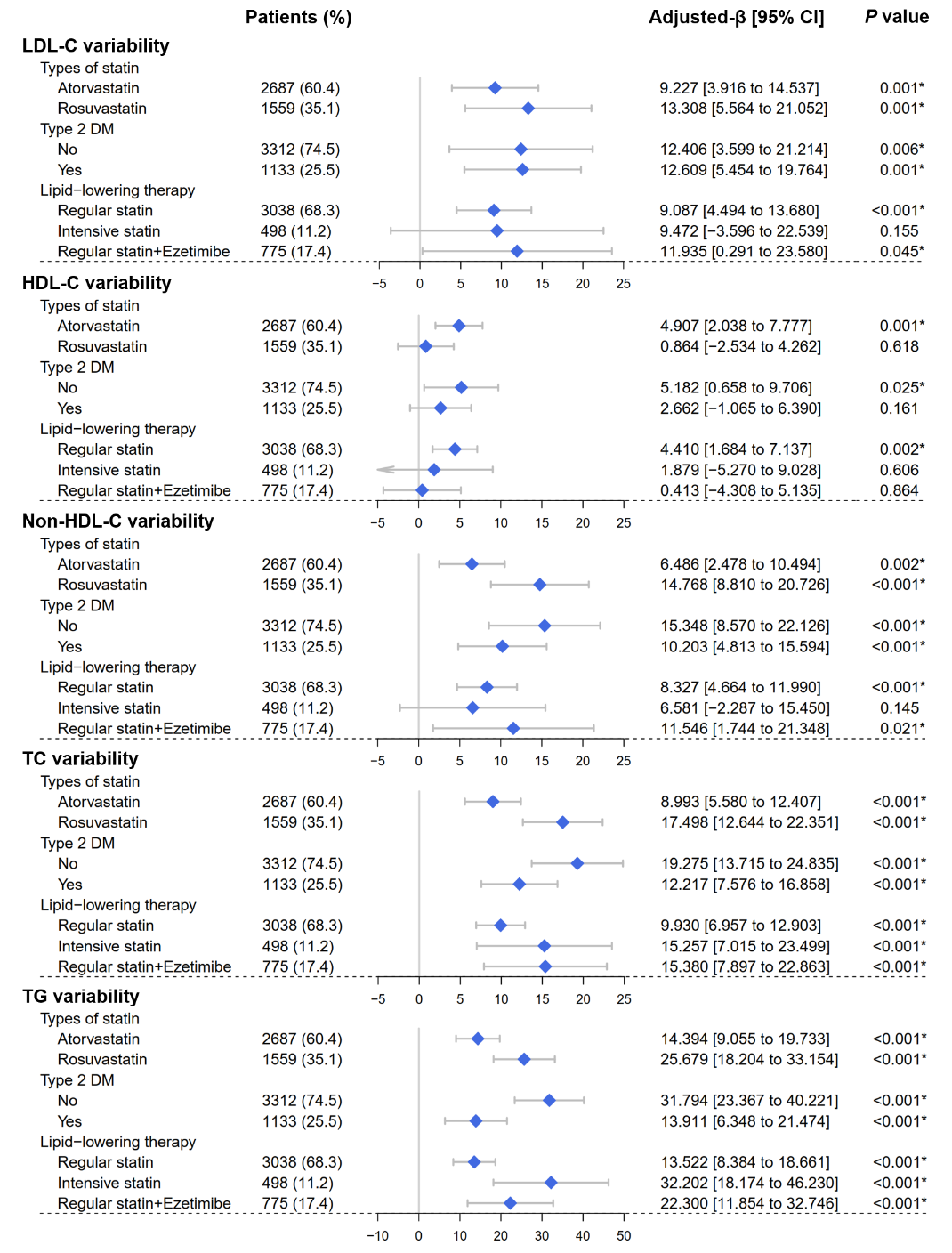


Forest plots depicted the effect of average follow-up HbA1c on the visit-to-visit variability of lipid profiles, including LDL-C, HDL-C, Non-HDL-C, TC, and TG. Lipid variability was represented by CV. Subgroups were determined according to Type 2 DM (yes or no), types of statins (atorvastatin or rosuvastatin), and lipid-lowering therapy strategy (regular statin/ intensive statin/ statin with ezetimibe). LDL-C indicates low-density lipoprotein cholesterol; HDL-C, high-density lipoprotein cholesterol; Non-HDL-C, non-high-density lipoprotein cholesterol; TC, total cholesterol; TG, triglyceride; DM, diabetes mellitus; CV, coefficient of variation. **P* <0.05.

**Figure S2.** Forest plot for lipid variability represented by SD


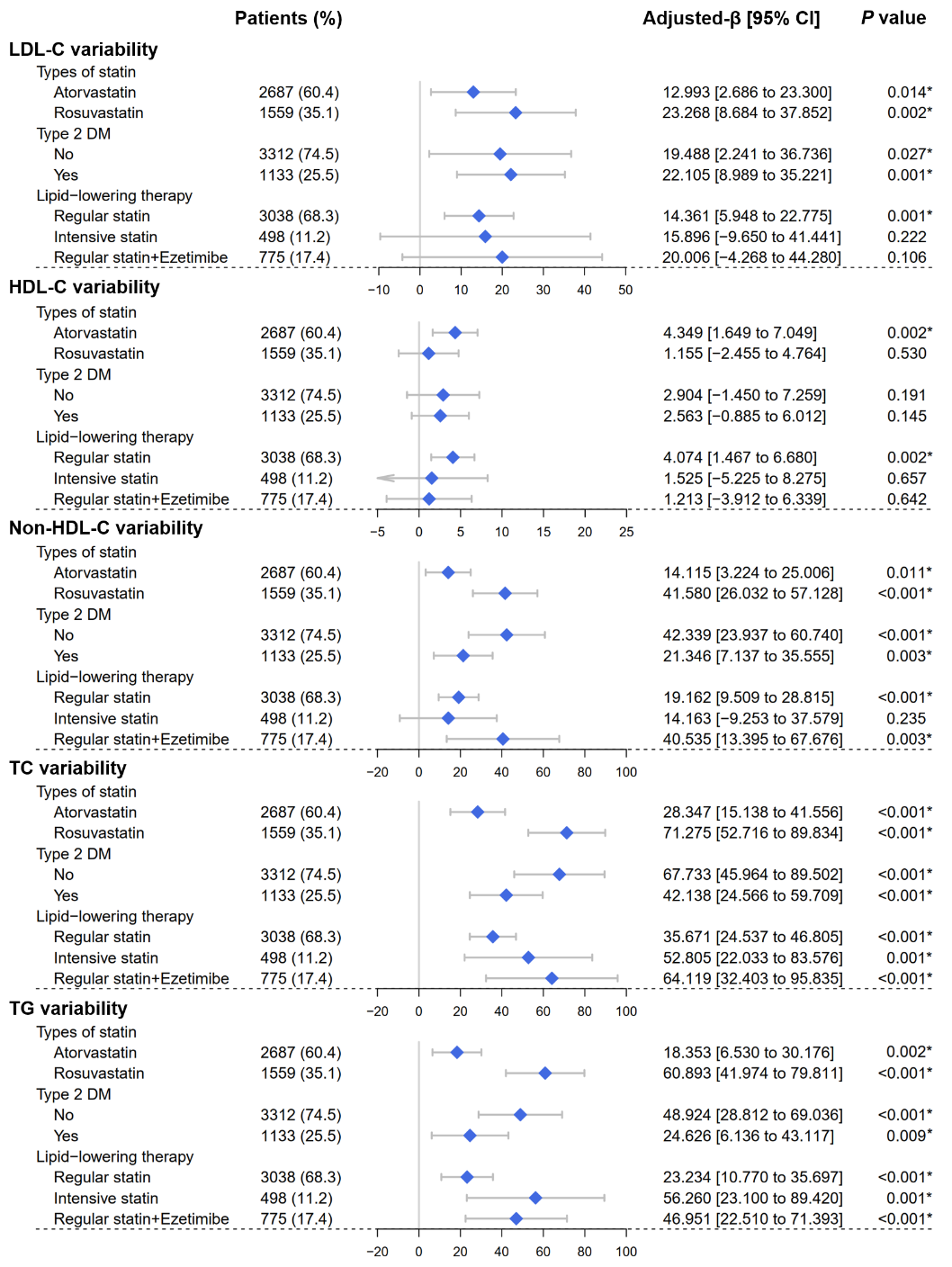


Forest plots depicted the effect of average follow-up HbA1c on the visit-to-visit variability of lipid profiles, including LDL-C, HDL-C, Non-HDL-C, TC, and TG. Lipid variability was represented by SD. Subgroups were determined according to Type 2 DM (yes or no), types of statins (atorvastatin or rosuvastatin), and lipid-lowering therapy strategy (regular statin/ intensive statin/ statin with ezetimibe). LDL-C indicates low-density lipoprotein cholesterol; HDL-C, high-density lipoprotein cholesterol; Non-HDL-C, non-high-density lipoprotein cholesterol; TC, total cholesterol; TG, triglyceride; DM, diabetes mellitus; CV, coefficient of variation. **P* <0.05.
